# Supplementary material for: “Someone who hates themself doesn’t come for their drugs”: Experiences of mental health along the HIV care continuum in South-Central, Uganda
Source: PLoS One. 2024 Oct 10;19(10):e0290809. doi: 10.1371/journal.pone.0290809 (PMC11466431; doi:10.1371/journal.pone.0290809)

Approved: 06July2020
IRB No.: 9695/MOD245

In-depth interview guide: people living with HIV
Version 2.0, 02 July 2020

Thank you again for agreeing to participate. I am going to ask you some questions about experiences of people living with HIV in Rakai, particularly related to emotional problems. I welcome you to speak freely; if you think of something you want to share that I have not asked about, please let me know. You do not have to answer any question you do not want to and you can stop at any time. Do you have any questions?

1. **Listing exercise:** *First I am going to start by asking you about problems that are experienced by people who have HIV in this community. There are no right or wrong answers – please list anything that comes to mind.*
    a. What are the main problems that affect people who have HIV in this community? *[Interviewer: write down all terms to refer back to for following questions]*
    For each problem listed:
        i. How can you tell when people have this problem?
        ii. How does this problem affect people living with HIV?
            1. Probe: How does having this problem affect how people living with HIV behave or act?
            2. Probe: How does having this problem affect how people living with HIV think of feel?
        iii. Does this problem only affect people living with HIV and why?
            1. Probe: If no – does this problem affect people with HIV differently than people who do not have HIV? How?
        iv. What is the cause of this problem?
        v. Where do people living with HIV seek care for this problem?
        vi. What can you do to treat this problem?

    b. *I know that people living in Rakai, whether they have HIV or not, may experience a number of problems or challenges like lack of jobs, not enough money, or different types of sickness like malaria. I am interested in problems that impact people who have HIV, and have to do with emotions, feelings and thinking.* What are the problems that people living with HIV experience that are related to emotions, feeling, or thinking? *[Interviewer: write down all terms to refer back to for following questions]*
    For each problem listed:
        i. How can you tell when people have this problem?
        ii. Does this problem only affect people living with HIV and why?
            1. Probe: If no – does this problem affect people living with HIV more or less than people who do not have HIV? How?
        iii. What is the cause of this problem?
        iv. How does this problem affect people living with HIV?
            1. Probe: How does having this problem affect how people living with HIV act?

2. Probe: How does having this problem affect how people living with HIV think or feel?
3. Probe: How does this problem affect how other people act towards people with HIV?

v. How do people living with HIV seek care or get treatment for this problem?
vi. What can be done to treat this problem?

2. **Timeline exercise.** *Next I am going to ask you about the experiences people living with HIV might have at different timepoints. I am going to use this timeline [show timeline to participant] to go through the next questions. You may not know about experiences at all points on the timeline, and that is fine. I am going to write on the timeline to take note of what you are saying you as you talk. Please feel free to point to or use the timeline to explain things to me as we talk.*
   a. Starting from the very beginning of the timeline, before someone receives their HIV diagnosis, and working up to the end of the timeline, please tell me about problems people who have HIV experience before, during, and after the different points along the timeline.
      i. Probe: Do people living with you experience any of the problems we have talked about related to emotions, feeling, or thinking at any points along the timeline? Where?
         1. Probe: If yes, where on the timeline do these occur?
         2. Probe: If yes, how long do these problems last for?
         3. Probe: If yes, what are the symptoms or feelings (emotional or physical) that people experience?
   b. [For any problems mentioned not previously discussed]: Please describe this problem to me.
         1. Probe: What do you think causes the problem to happen?
         2. Probe: Please describe the symptoms or feelings.
         3. Probe: Please show me on the timeline when or where the problem might start and end (if relevant).
         4. Probe: Please describe any treatment that people with HIV might seek for the problem.
   c. Looking at the whole timeline, do any of the problems we have discussed related to emotions, feeling, or thinking happen more or less or are better or worse at different points on this timeline?
      i. Probe: If yes, please describe why and how.
      ii. Probe: If no, why?
   d. Please share with me if there any problems that you think affect people living with HIV at different points on this timeline that we have not discussed.

3. **Impacts on daily life.** *Lastly, I am going to ask you about what happens to people living with HIV who experience the problems we have discussed. I am interested specifically in the problems that are related to emotions, feeling, or thinking that we have already discussed.*
    a. Please describe for me tasks and activities people living with HIV regularly do to care for themselves and their families. This could include things like going to work, doing household chores, or going to clinic appointments.
    b. For each problem identified as an emotional or mental health-related problem [from the listing or timeline activities]:
        i. For people who experience _________ problem, please describe how this causes changes to their daily life?
            1. Probe: Are there differences in changes to daily life between men and women?
        ii. How does this problem affect daily activities? Please describe how daily activities are harder. Please describe if there are differences between men and women.
        iii. How does this problem impact HIV care?
            1. Probe: Does having this problem affect taking HIV medication or going to the clinic?
            2. Probe: Are there differences in changes to HIV care for men and women?
        iv. How does this problem change how people act or behave?
            1. Probe: Are there differences in actions/behaviors between men and women?
    c. Of the problems we have talked about today that are related to thinking, feeling, and emotions, which do you feel are most important?
        i. Probe: Which of the problems affects people living with HIV the most or has the biggest impact on their daily life?

4. **Recommendations for key informants.** *We are hoping to interview other people from this community who know a lot about or provide treatment or support to people living with HIV who are experiencing mental health, emotional, feeling, or thinking problems.* Can you think of anyone like this who might be a good person for us to interview? To protect your privacy, we will not say that it was you who suggested we talk to them. [Record any potential key informants on the separate document for key informant details]

5. **COVID-19/Coronavirus questions:**

    a. Can you describe for me the impact coronavirus/COVID-19 has had on mental health, emotions, feelings, and thinking specifically for people living with HIV?

b. Are there any new problems we have not discussed related to mental health, emotions, feelings, and thinking that people living with HIV are experiencing as a result of COVID-19/coronavirus?
c. Please describe how coronavirus/COVID-19 has impacted how people living with HIV experience the problems we've discussed [list three prioritized problems one by one].

Thank you for answering my questions.
Please let me tell you a few important things about the new coronavirus/COVID-19.

Coronavirus or COVID-19 is a highly infectious disease that can spread from person-to person through sneezing and coughing, and through close contact. Symptoms of COVID-19 can include flu-like symptoms such as fever, cough, sore throat, difficulty breathing, and body pains and weakness. Everyone is at risk.

Most persons with COVID-19 get well in about two weeks. However, some people can get very sick and can even die. To protect yourself and others, some suggestions from the MOH include maintaining a reasonable distance between yourself and someone else of at least 2 meters, avoiding contact with persons who show flu-like symptoms, covering your mouth and nose when sneezing or coughing, and regularly washing your hands with soap and running water.
For more information, call the Ministry of Health toll free line on: 919, 0800-100-066, 0800-203-033 and 0800-303-033 or send a free SMS to Ureport on 8500 or WhatsApp on 0770-818-139.

Thank you again for agreeing to participate. I am going to ask you some questions about your job as a health worker, mental health problems of people living with HIV in Rakai, the conditions people living with HIV present with, and how people living with HIV describe their mental health problems. I welcome you to speak freely; if you think of something you want to share that I have not asked about, please let me know. You do not have to answer any question you do not want to and you can stop at any time. Do you have any questions before we begin?

1. **Training/Background.** *I will start by asking you a bit about your training and work experiences.*
    a. What type of health work do you primarily do?
    b. What type of training do you have for providing care to people living with HIV who are experiencing problems related to emotions, feeling, or thinking or mental health?
    c. Please tell me about a time you thought you had a patient with problems related to emotions, feeling, or thinking or mental health.
        i. Probe: What problems did this patient have (please describe in detail)?
        ii. Probe: How did you come to learn about these problems?
        iii. Probe: How did you treat or manage this patient?

2. **HIV care continuum exercise.** *I know that you work with people who may be at different places in their experience with HIV, such as newly diagnosed, have been on treatment for a long time, or have stopped taking their treatment or are having a hard time taking their treatment. You may not know about patients at all points in their HIV care and treatment, and that is fine. I am interested in problems that impact people who have HIV, and have to do with emotions, feelings and thinking at different points in their care and treatment.*
    a. Starting from just before someone receives their HIV diagnosis and knows their HIV status, please tell me about problems that people living with HIV experience that are related to emotions, feeling, or thinking. *[Interviewer to refer to the timeline and work through each point on the timeline asking the following questions]*

b. Of the problems we have discussed *[interviewer repeat back problems if necessary]*, if you had to pick the two that you think are most important, which two would you choose?
c. For the two problems mentioned [*repeat questions for each problem*]:
    i. How can you tell when people have this problem?
    ii. Does this problem only affect people living with HIV and why?
        1. Probe: If no – does this problem affect people living with HIV more or less than people who do not have HIV? How?
    iii. What is the cause of this problem?
    iv. How does this problem affect people living with HIV?
        1. Probe: How does having this problem affect how people living with HIV act?
        2. Probe: How does having this problem affect how people living with HIV think or feel?
        3. Probe: How does this problem affect how other people act towards people with HIV?
    v. How do people living with HIV seek care or get treatment for this problem?
    vi. What can be done to treat this problem?
d. Do any of the problems we have talk about related to mental health, emotions, feeling, or thinking happen more or less or are better or worse at different timepoints we have discussed?
    i. Probe: If yes, please indicate the time points and why and how.
    ii. Probe: If no, please explain to me why not?

3. Mental Health. *I am going to ask you some more detailed questions about people living with HIV who experience the problems we have discussed. I am interested specifically in the problems that are related to mental health, emotions, feeling, or thinking that we have already discussed.*
a. How do people living with HIV talk about mental health problems, or emotional problems?
    i. Probe: Are people in Rakai comfortable or uncomfortable talking about mental health problems?
    ii. Probe: Do people keep their mental health or emotional problems a secret?
b. Who do people living with HIV talk about mental health problems, or emotional problems to?
c. How many patients who are people living with HIV do you treat or counsel in a month?
d. Out of these ___ patients, how many might have a mental health problem?
e. What signs, behaviors or symptoms do you notice or do people complain of that make you think they have a mental health problem?
f. In your opinion, how does experiencing an emotional or mental health problem impact the tasks and activities people living with HIV regularly do

to care for themselves and their families. This could include things like going to work, doing household chores, or going to clinic appointments.

g. What are the mental health problems, problems of the emotion, or the mind you most commonly see among people living with HIV you provide care to?
h. Please describe the type of care a patient experiencing a mental health problem receives in the current health system?
i. In your opinion, what could be done to improve the way patients who experience mental health problems are given care?
j. In your opinion, what could be done to improve the way patients who experience mental health problems are identified?

4. **COVID-19/Coronavirus questions:**

   a. Can you describe for me the impact coronavirus/COVID-19 has had on mental health, emotions, feelings, and thinking specifically for people living with HIV?
   b. Are there any new problems we have not discussed related to mental health, emotions, feelings, and thinking that people living with HIV are experiencing as a result of COVID-19/coronavirus?
   c. Please describe how coronavirus/COVID-19 has impacted how people living with HIV experience the problems we've discussed [list two prioritized problems].

5. **Recommendations for key informants.** *We are hoping to interview other people from this community who know a lot about or provide treatment or support to people living with HIV who are experiencing mental health, emotional, feeling, or thinking problems.* Can you think of anyone like this who might be a good person for us to interview? To protect your privacy, we will not say that it was you who suggested we talk to them. [Record any potential key informants on the separate document for key informant details]

For follow-up interviews only:

6. **Specific Mental Health Problems.** *We previously spoke to other people in this community, and they told us about specific problems related to mental health, emotions, feeling, or thinking that affect people with HIV. Based on what we have been told, we identified "___________" as the biggest problems that affect people living with HIV. I would like for you to tell me everything you can about these problems.*

7. **Problem 1:**
   a. Tell me about _____________.
   b. How can you tell if someone has this problem? *(Symptoms, behaviors, other problems)*

c. What causes __________?
d. Where do people living with HIV seek care for ___________?
e. What can you do to treat ________________?
f. Some people living with HIV experience ___________, while other people living with HIV do not. Why do you think that some people living with HIV get this problem, while others do not?
g. How does having __________ affect the life of someone living with HIV?
h. Does having __________ affect anything about a person's ability to take care of their HIV or themselves?
i. Does _________ affect people living with HIV differently depending on what they are experiencing, such as whether they were just diagnosed with HIV, are taking or not taking medication?

Repeat questions for up to 4 problems.

Thank you for answering my questions.
Please let me tell you a few important things about the new coronavirus/COVID-19.

Coronavirus or COVID-19 is a highly infectious disease that can spread from person-to person through sneezing and coughing, and through close contact. Symptoms of COVID-19 can include flu-like symptoms such as fever, cough, sore throat, difficulty breathing, and body pains and weakness. Everyone is at risk.
Most persons with COVID-19 get well in about two weeks. However, some people can get very sick and can even die. To protect yourself and others, some suggestions from the MOH include maintaining a reasonable distance between yourself and someone else of at least 2 meters, avoiding contact with persons who show flu-like symptoms, covering your mouth and nose when sneezing or coughing, and regularly washing your hands with soap and running water.
For more information, call the Ministry of Health toll free line on: 919, 0800-100-066, 0800-203-033 and 0800-303-033 or send a free SMS to Ureport on 8500 or WhatsApp on 0770-818-139.

Approved: 06July2020
IRB No.: 9695/MOD245

In-depth interview guide: people living with HIV
Version 2.0, 02 July 2020

Appendix 1 – Timeline Example
Participant ID

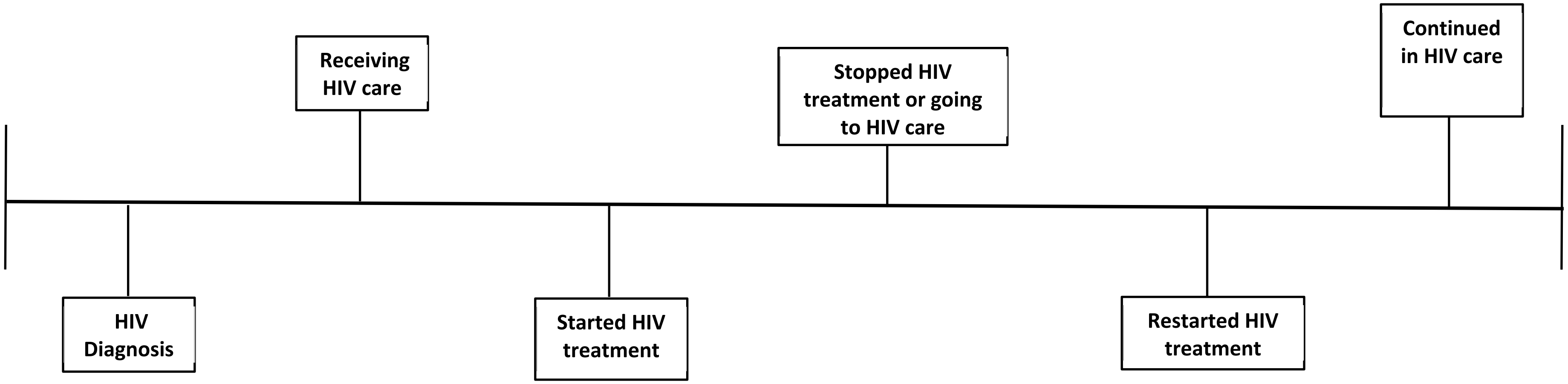

Supplement: S1 File — (PDF) [file pone.0290809.s001.pdf]
